# SOLiD™ Sequencing of Genomes of Clinical Isolates of *Leishmania donovani* from India Confirm *Leptomonas* Co-Infection and Raise Some Key Questions

**DOI:** 10.1371/journal.pone.0055738

**Published:** 2013-02-13

**Authors:** Neeloo Singh, Surendra Chikara, Shyam Sundar

**Affiliations:** 1 CSIR- Central Drug Research Institute, Lucknow, India; 2 Xcelris Genomics, Ahmedabad, India; 3 Institute of Medical Sciences, Banaras Hindu University, Varanasi, India; Tel Aviv University, Israel

## Abstract

**Background:**

Known as ‘neglected disease’ because relatively little effort has been applied to finding cures, leishmaniasis kills more than 150,000 people every year and debilitates millions more. Visceral leishmaniasis (VL), also called Kala Azar (KA) or black fever in India, claims around 20,000 lives every year. Whole genome analysis presents an excellent means to identify new targets for drugs, vaccine and diagnostics development, and also provide an avenue into the biological basis of parasite virulence in the *L. donovani* complex prevalent in India.

**Methodology/Principal Findings:**

In our presently described study, the next generation SOLiD™ platform was successfully utilized for the first time to carry out whole genome sequencing of *L. donovani* clinical isolates from India. We report the exceptional occurrence of insect trypanosomatids in clinical cases of visceral leishmaniasis (Kala Azar) patients in India. We confirm with whole genome sequencing analysis data that isolates which were sequenced from Kala Azar (visceral leishmaniasis) cases were genetically related to *Leptomonas.* The co-infection in splenic aspirate of these patients with a species of *Leptomonas* and how likely is it that the infection might be pathogenic, are key questions which need to be investigated. We discuss our results in the context of some important probable hypothesis in this article.

**Conclusions/Significance:**

Our intriguing results of unusual cases of Kala Azar found to be most similar to *Leptomonas* species put forth important clinical implications for the treatment of Kala Azar in India. *Leptomonas* have been shown to be highly susceptible to several standard leishmaniacides *in vitro*. There is very little divergence among these two species viz. *Leishmania* sp. and *L. seymouri*, in terms of genomic sequence and organization. A more extensive perception of the phenomenon of co-infection needs to be addressed from molecular pathogenesis and eco-epidemiological standpoint.

## Introduction

The family of Trypanosomatidae belongs to the order of Kinetoplastida protozoa. These are the most primitive organisms in eukaryotic evolution to have mitochondria and peroxisomes [1]. These parasites have different life cycles that involve one host (monoxenous) e.g. *Leptomonas*, or two hosts (heteroxenous) e.g. *Leishmania.* The latter involves an invertebrate that acts as a vector between other vertebrates or plants. During differentiation in the insect gut and in culture, these kinetoplastid protozoans appear as promastigotes (*Leptomonas* and *Leishmania*), and amastigotes (*Leishmania*), which develop in the mammalian host macrophage and causes disease. The ancestral form of *Leishmania* was *Leptomonas*, an organism living solely in the invertebrate host and transmitted by the ingestion of resistant forms (cysts) expelled with the excreta of the host [2]. Unlike *Leptomonas, Leishmania* produces no resistant cysts capable of development in the invertebrate host and has adapted to a life cycle alternating between invertebrate and vertebrate hosts [3], [4].

*Leishmania*, a trypanosomatid protozoan parasite of humans, causes a wide spectrum of clinical disease referred to as leishmaniasis. Leishmaniasis represents a global health problem and is prevalent in Europe, Africa, Asia and the Americas; up to twenty million people are infected and half a million are affected by the lethal VL (www.dndi.org). In the Indian sub-continent, visceral leishmaniasis or Kala Azar (KA) as it is popularly known, is caused by *Leishmania donovani* and transmitted by the sandfly of genus *Phlebotomus argentipes.* One hundred fifty million people are living with the risk of VL in the Indian subcontinent (India, Nepal, and Bangladesh) [5].

Indian Kala-azar (VL) has a unique epidemiological feature of being anthroponotic; human are the only known reservoir of infection [6]. Localized cutaneous leishmaniasis (LCL) in India is mostly due to *Leishmania tropica* and is endemic in the deserts of Rajasthan [7], [8]. Few cases of LCL among travelers have been documented in other Indian states such as Kerala [9], Assam, and Haryana, which are not disease-endemic areas [8]. A recognized endemic focus of leishmaniasis in Satluj river valley in Himachal Pradesh has been reported [10]. This endemic focus of leishmaniasis appears peculiar where localized cutaneous leishmaniasis (LCL) co-exists with visceral leishmaniasis (VL), and *Leishmania donovani* is predominant pathogen for LCL whereas only a few cases have been due to *Leishmania tropica*. *P. longiductus* a known vector for *L. infantum*, is the main vector in this endemic focus. *L. donovani infantum* causing both cutaneous and visceral leishmaniasis and K39 seroprevalence in dogs (known reservoir for *L. infantum)* have been reported for this region [11].

During 1993–1994, scientists from developing and developed countries planned and initiated a number of parasite genome projects and several consortiums for the mapping and sequencing of these medium sized genomes were established. Genomes of three *Leishmania* species, which were cultivated in the laboratory (*L. major, L. infantum, L. braziliensis* and *L. mexicana*) have been sequenced [12]. The New World parasite *L. braziliensis* is the causative agent of mucocutaneous leishmaniasis, whereas the Old World species *L. major* and *L. infantum*, which are present in Africa, Europe and Asia, are parasites that cause cutaneous and visceral leishmaniasis, respectively [4]. It has been reported that *L. donovani* is genetically distinct from *L. infantum* [13]. To add to this plethora of knowledge, we undertook whole genome sequencing of clinical isolates of *L. donovani* believed to be causing Kala Azar in India. The genome analysis can provide insights into the functional characteristics of the visceral manifestation of the disease and also provide an avenue into the biological basis of parasite virulence in the *L. donovani* complex prevalent in India in comparison with the other species of *Leishmania* sequenced. The genome of *L. donovani* from India has so far not been sequenced.

## Materials and Methods

### Clinical Isolates

During the year 1998–2000, resistance to the widely used antimonial drug sodium antimony gluconate (SAG) had reached alarming heights in India [14]. At this time we had cultivated many isolates collected from the eastern region of India. Clinical isolates from confirmed patients of Kala Azar from endemic zone of Bihar and Uttar Pradesh were collected as splenic aspiration performed by our authorized clinical collaborator and co-author Dr Shyam Sundar, with prior written consent of the patients. Institutional Review Board (Banaras Hindu University, Varanasi) approved the study. The diagnostic criteria for Visceral Leishmaniasis (VL) were the presence of LD bodies (Leishman Donovan) in splenic aspirations performed and graded as per standard criteria [15]. Isolate 39, used for whole genome sequencing in this study, was isolated on 28.05.2000 from Muzaffarpur, Bihar from splenic aspirate of a patient who did not respond to SAG therapy, whereas isolate 2001, isolated on 01.02.2000, from Balia, Uttar Pradesh, responded to SAG therapy. Isolate Ld BHU 1095, responsive to amphotericin B was collected relatively recently from Muzaffarpur in Bihar on 31.07.2010. Splenic aspirates were collected and adapted to culture as described [16]. The virulence and level of susceptibility or resistance of these isolates was confirmed *in vitro* and *in vivo*, by infection in experimental animals as described [17]. Species identity of these promastigotes was confirmed to be similar to the *donovani* in the sequence based RFLP of their single-copy protein-coding gene, N-acetylglucosamine-1-phosphate transferase [18]. The isolates used in our study have also been the subject of various studies in leishmaniasis by many groups worldwide. In our hands (16,17) and also with others who have worked and published on these two isolates, they served as an excellent model of visceral leishmaniasis, producing typical clinical outcome consistently, including invariable death and splenic LD loads upon infection in hamsters of up to 10^10^ per heavily infected spleen.

### Solid DNA Sequencing

The genomic DNA of the parasites was isolated using the QIAamp DNA isolation kit (Qiagen, Catalog No. 51104). Sequencing runs were done using cycled ligation sequencing on a SOLiD™ Next Generation Sequencer (Applied Biosystems, India). Mate pair library approach was utilized thus generating data for two tags denoted as F3 and R3. Two segments of the Quad slide format were utilized to generate the data for each sample. Approximately, 5 ug of purified genomic DNA was sheared for a mate paired library with insert size between 1.5–2 Kb The blunt-ended ligation of sheared DNA was carried out to convert DNA with damaged or protruding ends to phosphorylated, blunt-ended DNA. After that LMP CAP ligation was performed to add the LMP CAP adaptors to the sheared, end repaired DNA. Size-selection was performed after CAP adaptor ligation to remove unbound CAP adaptors. The sheared DNA was ligated to LMP CAP adaptors and then be circularized with a biotinylated internal adaptor. Nick translation was carried out to translate the nick into the genomic DNA region. Then enzymatic digestion was carried out followed by blunt-ended ligation. P1 and P2 adaptors were ligated to the ends of the end-repaired DNA. Nick translation of the ligated, purified library was performed. The library was amplified using Library PCR Primers and was run on an SOLiD™ Library Size selection gel. The library size ranging between 275 bp to 350 bp was finally selected for template bead preparation. For the clonal amplification of library emulsion PCR was performed. After emulsion PCR, bead wash was performed and enrichment of the template beads was carried out. Modification of 3′ ends was carried out for deposition of beads on glass slide. Quantification of beads using Nanodrop spectrophotometer was done. Work flow analysis (WFA) was carried out to confirm the quality of the beads.

### Mapping and Assembly

The mapping module of the standard Resequencing workflow of BioScope v 1.3 software was utilized which has an algorithm designed on seed and extend approach of mapping. A quality value is associated with each alignment. The quality value estimates the probability that the alignment is correct. Output of pairing was a BAM file (Binary format for Sequence Alignment Map) which stores the read alignment in coordinate order. The colorspace reads from SOLiD sequence were aligned to the reference genomes. Reads from each of the isolates were mapped to the reference genome separately. Reads unmapped were identified. Reads with low complexity characteristics, containing homopolymer tract, at least four repeats of the same di nucleotide or tri nucleotide in a row, were removed from the data set before further analysis. Although these reads maybe representing true genomic regions, however, the inherent difficulty in assigning them to a particular genomic region limits their value. This is an inherent problem with short read data of SOLiD sequencing system. Summary of pairing results sequencing quality parameters were ascertained by Samtools v0.1.6. De Novo Analysis V 2.0 software was utilized and assembly was performed by velvet assembler. The estimation of genome size by k-mer frequency distribution analysis was done.

As a first step, tags (F3 and R3) of each of the three genomes were mapped to the reference genome *Leishmania infantum* (LinJ), which was downloaded from the following ftp site: ftp://ftp.sanger.ac.uk/pub4/pathogens/Leishmania/infantum/V52010/artemis/EMBL/Linfantum/1/with a total genome length incl. Gaps = 32,126,170 bp. The mapping module of the standard Resequencing workflow of BioScopeTM v1.3 software was utilized which has an algorithm designed on seed and extend approach of mapping. A quality value is associated with each alignment. The quality value estimates the probability that the alignment is correct. Output of pairing was a BAM file (Binary format for Sequence Alignment Map) which stores the read alignment in coordinate order.

### Phylogenetic Analysis on the Basis of GP63 Gene

GP63 gene sequences from Ld39, BHU1095 and Ld2001 were obtained from annotated assembled contigs. The GP63 gene sequences of *L. infantum* and *L. donovani* Nepal strain Ld_BPK282A1 were obtained from Ensembl and *Leptomonas* was identified using homology based method. A phylogeny of these strains was carried using GP63 gene sequences by multi-sequence alignment using CLC genomics workbench v5.1 with gap open cost 10, gap penalty score of 1. Phylogenetic tree was measured by using the bootstrap method with 1,000 replicates; for more computationally intensive ML trees, we used 100 bootstrap replicates by UPGMA methods.

### Phylogenetic Analysis on the Basis of ITS Gene

ITS gene sequences from Ld39, BHU1095 and Ld2001 were obtained from annotated assembled contigs. The ITS gene sequences of *L. infantum* were obtained from Ensembl, *L. donovani* Nepal strain Ld_BPK282A1 and *Leptomonas* was identified using homology based method. A phylogeny of these trains was carried using ITS gene sequences by multi-sequence alignment using CLC genomics workbench v5.1 with gap open cost 10, gap penalty score of 1. Phylogenetic tree was measured by using the bootstrap method with 1,000 replicates; for more computationally intensive ML trees, we used 100 bootstrap replicates by UPGMA methods.

### Accession Codes

This Whole Genome Shotgun project has been deposited at DDBJ/EMBL/GenBank under the accession

ANFN00000000. The version described in this paper is the first version ANFN01000000 for *Leishmania donovani* Ld 39;

ALJU00000000, the version described in this paper is the first version, ALJU01000000 for *Leishmania donovani* Ld 2000;

ANAF00000000, the version described in this paper is the first version, ANAF01000000 for *Leishmania donovani* BHU 1095.

## Results

### SOLiD Sequencing Reads

The total number of 35-bp reads for each isolate were Ld 2001 −19×10^7^, Ld 39 −20.4×10^7^, BHU 1095 −29.7×10^7^ yielding approximately 200 fold coverage for each of the three genomes with the assumption that all the data was usable (Table 1). Using De Novo Analysis V2.0 software, assembly was performed by velvet assembler at a kmer of 31, full set of data was utilized for this analysis. Results of final outcome of assembly in contig and scaffold file in base space was obtained (Table 2).

**Table 1:** Mapping statistics of SOLiD mate pair reads to reference genome *Leishmania infantum* (LinJ).

	Sample Ld 2001		Sample Ld 39		Sample Ld BHU 1095	
	F3 Tags/Reads	R3 Tags/Reads	F3 Tags/Reads	R3 Tags/Reads	F3 Tags/Reads	R3 Tags/Reads
Total Reads	95,150,090	95,150,090	102,142,421	102,142,421	148,702,153	148,702,153
Total Mapped Reads	2,610,988	2,987,733	2,918,610	3,196,042	12,803,914	12,803,914

**Table 2:** De Novo Assembly Statistics.

Assembly Stats	Ld 2001	Ld 39	Ld BHU 1095
Number ofcontigs/scaffolds	14518	16389	18232
Total genomelength (bp)	27466456	23799529	16806104
Average contigs/scaffold length	1891	1452	921
Contigs/Scaffold N50	3370	1773	961
Max. contigs/scaffoldsize (bp)	26366	11755	4772
Min. contigs/scaffoldsize (bp)	200	500	500

### Comparison of SOLiD Sequencing Reads to Reference *Leishmania* Genomes

Unexpectedly, only 2–3 percent of reads of Ld 2001 and Ld 39 mapped to LinJ genome. From total of 2×297 M reads obtained for Ld BHU 1095, 26 M reads mapped to LinJ genome which is ∼9 percent of the total reads (Table 1). Despite approximately 200 fold coverage based on the raw number of reads obtained for each isolate genome, some number of unmapped reads were also obtained which is a limitation of short read technology. There is also a possibility that these maybe derived from regions of low complexity in the sequenced genomes. Remaining umapped reads which are not of low complexity, could be truly unique sequences or errors in the sequencing system. In an attempt to identify between these two possibilities, mapping the reads in SOLiD color space using MAQ was employed to identify orthologs. Largest number of matches were found with genomic sequence of *Leptomonas seymouri.*

*L. donovani* Nepal isolate [19] BPK282AI reference genome was downloaded from site: http://www.ebi.ac.uk/embl (acession number FR799588-FR799623) with a total genome length incl. Gaps of 32,444,968 bp. The genome coverage of BHU_1095, Ld_39 and Ld_2001 was computed against *Leptomonas* and *L. donovani* Nepal strain Ld_BPK282A1 by reference assisted assembly. The BHU_1095, Ld_39 and Ld_2001 reads were aligned with *Leptomonas* and *L. donovani* Nepal strain Ld_BPK282A1 genome using Bioscope v1.3. The percentage of reads aligned on *Leptomonas* were 31.02% for BHU_1095, 67.11% for Ld_39 and 65.70 for Ld_2001 respectively. Similarly, the percentage of reads mapped on L. donovani Nepal strain Ld_BPK282A1 were 0.50% for BHU_1095, 0.49% for Ld_39 and 0.53% for Ld_2001 respectively. The genome coverage with respect to *Leptomonas* is around 95% and for *L. donovani* Nepal strain Ld_BPK282A1 its around 0.5% for all the samples.

From the Tables 3 and 4, it’s clear that raw reads of BHU1095, Ld_39 and Ld_2001, covers more than 90% of the *Leptomonas* genome whereas less than 1% of the Ld_BPK282A1 genome (*L. donovani* Nepal strain) indicating that there is a presence of *Leptomonas* and *Leishmania,* and *Leptomonas* is predominant.

**Table 3:** Statistics of genome coverage in percentage and bases with *Leptomonas* and *L. donovani* Nepal isolate Ld_BPK282A1.

Reference Description	Bases covered			Genome coverage		
	BHU_1095	Ld_39	Ld_2001	BHU_1095	Ld_39	Ld_2001
**Leptomoas**	53426355	53551698	53700910	95.38%	95.60%	95.87%
**Ld_BPK282A1**	166098	161811	174694	0.50%	0.49%	0.53%

**Table 4:** Mapping statistics of reads with *Leptomonas* and *L. donovani* Nepal isolate Ld_BPK282A1.

Reference Description	No. of reads mapped(paired)		
	BHU_1095	Ld_39	Ld_2001
**Leptomoas**	42632516	50109373	44858095
**Ld_BPK282A1**	179637	190865	231275

It has been noticed that with very high coverage such as used here, at times the data actually becomes harder to interpret as the number of chimeric clones and errors start to generate a large amount of noise. Therefore we used a subset of the data, 10 million reads were selected from the total data set of sample BHU 1095, this being the most recent of the clinical isolates to have been collected from patient in endemic region, and denovo assembly was performed using velvet 1.1.04 at kmer 25 and kmer31. The contigs from both the kmers were merged and final assembly was performed using CAP3. The assembly contained 1,27,259 contigs (N50 contig size of 159 bp) and total genome length of 19.3 Mb. For downstream analysis we proceeded with 1,27,259 contigs. The cut-off of 500 bp was applied for filtration of contigs resulting in 1485 contigs. BLASTN against nr database was carried out for 1485 contigs, out of which 73 contigs had a BLAST result. 44 contigs out of 73 had hit against *Leishmania.*

Genome sequence data for Ld BHU 1095 was also generated on HiSeq2000 using Paired-end 2×100 bp. Reads generated were 1,00,000. One lac (1×50) paired end reads were mapped on *Leishmania infantum* reference genome and *Leptomonas*, using GS Mapper. Reads of sample Ld BHU 1095 mapping to *L. infantum* 2.9%; *Leptomonas* 89.7%; *L donovani* Nepal isolate 19.37%.

**Figure 1:**
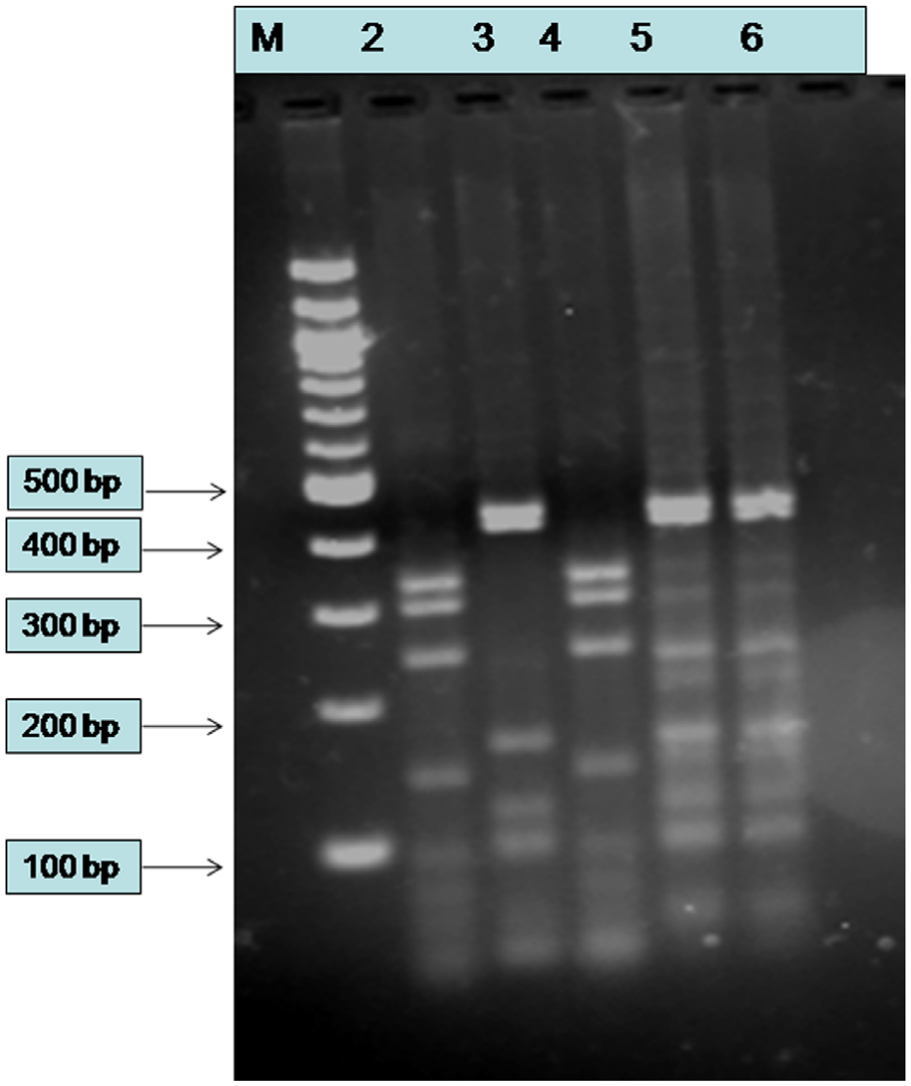
Agarose gel analysis of hsp70 PCR-RFLP from different parasite isolates. The PCR products of 1420 bp were digested with the restriction enzyme HaeIII. Digested products were separated on 3% Small Fragment Agarose (Sigma,India). Lane M is a 100 bp Ladder (New England Biolabs, UK). Lanes 2- confirmed *L. donovani* (BHU-744), Lane 3: confirmed *Leptomonas* (BHU-154). Lane 4 is a BHU-1095 showing *L. donovani* signature. Lanes 5 and 6: BHU 39 and BHU 2001 respectively.

The data from different platforms concluded that all the three samples were having a major portion of *Leptomonas* genome and small percentage of *Leishmania* genome. We then carried out Hsp70 PCR-RFLP [20] and confirmed mixed infection, signals of both *Leptomonas* spp. and *Leishmania donovani* were obtained in the two isolates Ld 2001 and Ld 39 whereas Ld BHU 1095 showed *L*. *donovani* pattern (Fig. 1). It is clear from this gel that Ld 2001 and Ld 39 contained a mixture of *Leptomonas* and *Leishmania* DNA (with considerably more of the former). Both these isolates did not appear to be a perfect match for either *Leptomonas* or *Leishmania* DNA, since they contained two additional bands at ∼200–250 bp found in neither BHU-154 nor BHU-744. They also lacked the bands found at ∼50 bp. BHU-1095 contained only *Leishmania* restriction pattern and not *Leptomonas.* However, whole genome sequencing of BHU 1095 revealed only ∼20% similarity to *Leishmania* and rest attributed to *Leptomonas,* indicating that traditional multilocus typing methodology is not indicative of revealing complete genetic structure.

Presently, we are also undertaking sequencing of amastigote proteins of our samples. Preliminary experiments using LC MALDI of Ld 2001 identified 100 proteins with *L. major* database (unpublished results). Possibly, these are *Leptomonas* proteins that have peptides similar enough to *L. major.* When the search was done using *Leptomonas* proteins, (predicted from our assembled genome of Ld 2001, which has been confirmed by us through whole genome sequencing, to be *Leptomonas seymouri* like, we obtained 236 peptide hits which is much more higher than what we got in *L. major.* This information will prove helpful subsequently for validation of the *Leptomonas* gene prediction/annotation on our samples.

The possibility of contamination of our cultures is highly unlikely. There has never been any *Leptomonas* culture in our or clinical collaborator’s laboratory. The original isolates itself must be co-infection. Using PCR-RFLP, co-infection of *Leptomonas* in splenic aspirates of kala azar patients in India, is also being reported by many other groups too [21], [22], however, as our results point out in the case of isolate BHU 1095, PCR RFLP may not be foolproof in differentiating between the genera genera of *Leptomonas* with *Leishmania*. Using 18S rRNA gene sequencing, it has been reported [20] that 7% isolates of Kala Azar were similar to *Leptomonas* sp in Bihar region. However, we are of the opinion that 18S rRNA gene sequencing is not the absolute indication of correct detection of *Leptomonas* sp. parasites in clinical isolates of Kala Azar patients as it has been well established that overall DR structure [the maxicircle control region, also termed divergent region (DR)] is quite conserved in the species of *Leishmania –Leptomonas* group (the slow evolving 18S rRNA sequences). In this aspect our study is of prime importance in unequivocally establishing by whole genome sequencing the presence of *Leptomonas* in clinical isolates of Kala Azar in India. Our study has made an important contribution in generating whole genome sequencing which can be developed by researchers into interesting evolutionary biology analysis.

**Figure 2:**
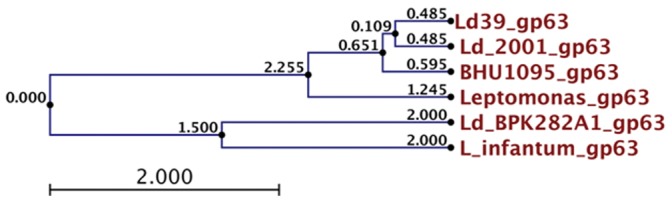
Phylogenetic relationships among strains of the *L. donovani and Leptomonas* complex inferred by UPGMA method of the nucleotide sequences of the GP63 genes.

The genomes of various *Leishmania* parasites contain tandemly arrayed genes encoding an abundant 63-kDa surface glycoprotein called GP63 and present in all insect and plant trypanosomatids. Even though the three clinical isolates of *Leishmania donovani* used in the present study have major portion of the *Leptomonas* genome, yet using these isolates, the taxonomic status of *L. donovani* and *L. infantum*, as discrete species has been established by phylogenetic analysis (Fig. 2; Table 5). The phylogenetic analysis of GP63 genes shows that Ld39, Ld2001, BHU1095 are closely related to *Leptomonas*. *Leishmania infantum* and *L. donovani* Nepal strain Ld_BPK282A1 fall in same clade.

**Table 5:** Designation and characteristics of GP 63 genes and strains used in this study.

GP63	
Taxa	Accession number EMBL/NCBI/Contig_IDs
Ld 2001_gp63	399500213
	399502729
	399502737
	399503298
	399503299
	399503897
	399504021
	399504638
	399505322
	399506275
	399506280
	399507247
	399507249
	399508864
	399510205
	399510518
	399510602
	399510705
	399510751
	399510752
	399510947
	399511516
	399511596
	399512213
	399512449
	399512573
	399512951
	399513018
	399513027
	399513206
Ldonovani_BPK282A1	LDBPK_100510
	LDBPK_100520
	LdBPK_280600
	LdBPK_280610
	LdBPK_312040
Leptomonas	>NODE_16451-0.947-1
	>NODE_18931-0.2081-0
	>NODE_18931-0.2081-1
	>NODE_34334-0.1195-28
	>NODE_40634-0.206-43
	>NODE_40634-0.206-45
	>NODE_43570-0.1019-25
	>NODE_46564-0.1189-16
	>NODE_49117-0.518-30
	>NODE_50764-0.655-84
	>NODE_50908-0.2398-28
	>NODE_52179-0.1261-100
	>NODE_52179-0.1261-99
	>NODE_60985-0.3035-55
Leishmania infantum	
	LINJ_10_0530
	LINJ_10_0510
	LINJ_31_2040
	LINJ_10_0500
	LINJ_10_0490
	LINJ_10_0520
	LINJ10.0790
	LINJ_28_0610
	LINJ_28_0600
	LINJ28.0580
	LINJ28.3180
BHU1095	
	contig_153
	contig_167
	contig_466
	contig_1286
	contig_2242
	contig_2456
	contig_2506
	contig_2723
	contig_3083
	contig_3912
	contig_4178
	contig_4629
	contig_4865
	contig_4971
	contig_5352
	contig_5494
	contig_6288
	contig_6300
	contig_6541
	contig_6621
	contig_7021
	contig_8550
	contig_10874
	contig_11736
	contig_12421
	contig_12771
	contig_13714
	contig_13983
	contig_14146
	contig_14343
	contig_14377
	contig_14709
	contig_14788
	contig_15481
	contig_16073
	contig_16466
	contig_17609
	contig_17869
	contig_17924
LD_39	
	contig_7416
	contig_7441
	contig_8106
	contig_103
	contig_9808
	contig_1128
	contig_1164
	contig_1294
	contig_11517
	contig_11541
	contig_12419
	contig_12698
	contig_12699
	contig_12702
	contig_13126
	contig_13516
	contig_14137
	contig_1601
	contig_14878
	contig_15648
	contig_15897
	contig_16264
	contig_2198
	contig_2354
	contig_353
	contig_3645
	contig_455
	contig_502
	contig_4961
	contig_5341
	contig_5407
	contig_617
	contig_5972
	contig_6290

**Figure 3:**
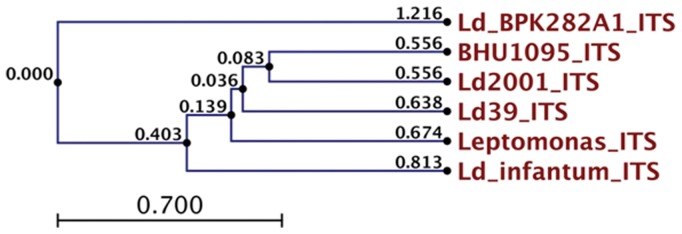
Phylogenetic relationships among strains of the *L. donovani* and *Leptomonas* complex inferred by UPGMA method of the nucleotide sequences of the ITS1 and ITS2 regions.

We also analyzed genetic diversity based on the amplification of the internal transcribed spacers (ITS), located within the rRNA gene array. ITS sequences are used to generate information useful for phylogenetic reconstruction and molecular evolution studies. It is clear from the ITS phylogenetic tree (Fig. 3; Table 6) that Ld 2001, Ld 39 and Ld BHU 1095 are closely related. *L. infantum* is most distantly related from the three. The phylogenetic analysis of ITS genes based on the distance (∼0.5) shows that Ld39, Ld2001, BHU1095 are closely related to *Leptomonas*. *Leishmania infantum* and *L. donovani* Nepal strain Ld_BPK282A1 belong to different clade of *Leishmania.*

**Table 6:** Designation and characteristics of ITS genes and strains used in this study.

Leishmania_infantum_ITS1_2	
	JX448543.1
	JX448541.1
	JX448539.1
	JX448537.1
	JX448535.1
	JX448546.1
	JX448544.1
	JX448542.1
	JX448540.1
	JX448538.1
	JX448536.1
	JX289879.1
	JX289853.1
	JX289880.1
	JX289852.1
	HQ535858.1
	FN398341.2
	FN398342.1
	JQ776643.1
	DQ182535.1
	HQ830353.1
	JQ362410.1
	GU045592.1
	FN398343.2
	GQ444144.1
	GU591397.1
	FJ940893.1
	FJ940891.1
	FJ940892.1
	FJ555210.1
	EU604812.1
	EU604810.1
	EU810777.1
	FJ555211.1
	FJ555209.1
	EU810776.1
	EU326227.1
	FJ497004.1
	EF653268.1
	GQ367488.1
	GQ367486.1
	GQ367487.1
	GQ332359.1
	EU680963.1
	EU637915.1
	EU680962.1
	FM164420.1
	FM164418.1
	FM164416.1
	FM164419.1
	FM164417.1
	AJ634370.1
	AJ634364.1
	AJ634362.1
	AJ634354.1
	AJ634352.1
	AJ634350.1
	AJ634348.1
	AJ634346.1
	AJ634344.1
	AJ634342.1
	AJ634340.1
	AJ634371.1
	AJ634369.1
	AJ634363.1
	AJ634361.1
	AJ634355.1
	AJ634353.1
	AJ634351.1
	AJ634349.1
	AJ634347.1
	AJ634345.1
	AJ634343.1
	AJ634341.1
	AJ634339.1
	AJ000288.1
	AJ000303.1
	AJ000295.1
	AJ000289.1
Leptomonas	
	>NODE_3422-0.590-0
	>NODE_8222-0.589-0
Ld_2001	gi|399513055|gb|ALJU01000452.1|
	gi|399513471|gb|ALJU01000036.1|
BHU1092	
	contig_3530
LD_39	
	>contig_1060
	>contig_707
	>contig_33

## Discussion

The occurrence of *Leptomonas* along with *Leishmania* amastigotes in splenic aspirates of Kala Azar patients is a unique phenomena worth exploring. Srivastava et al [20] have attributed this to the immune system depression in the patient to explain the opportunistic parasitism by this trypanosomatid. We put forth certain questions which need investigation.

Flagellates of the family Trypanosomatidae fall into two natural groups. The primitive genera is *Leptomonas* which is confined to invertebrates, and the more advanced genera is *Leishmania* which uses both vertebrate and invertebrate host [23]. The monogenetic species *Leptomonas* are also known as “lower trypanosomatids” because the digenetic genera *Leishmania* are thought to have arisen from a monogenetic ancestor [24] and are parasitic in arthropods mainly in Insecta and Diptera [1]. The parasites are found in various sections of the alimentary tracts of infected insects, and transmission is assumed to largely follow contaminative pathways [2]. The morphology of the insect-inhabiting stages of the pathogenic digenetic species resembles that of the monogenetic species. *Leptomonas* shares a promastigote stage of development with *Leishmania*. These lower trypanosomatids are characterized by ease of cultivation and less fastidious nutritional requirements than *Leishmania*. Promastigotes of the clinical isolates in this study transformed into amastigotes and survived in cultured macrophages as well as in experimental hamsters [16], [17]. Promastigotes of *Leptomonas costoris*, a kinetoplastid parasite of water striders, transformed into amastigotes but did not survive in cultured macrophages [25]. So does our study indicate that *Leptomonas* is influencing the pathogenesis of leishmaniasis? Is the distribution of *Leishmania* strains containing *Leptomonas* limited to specific eastern region of India, or occur elsewhere, also needs to be ascertained.

The results of our study put forth certain debatable issues. In India for VL in Bihar region, anthroponotic transmission with no intermediate host has been observed [26]. But our study now questions the fact that is VL or KA in India actually not anthroponotic as is believed till today? The source could be zoonotic, dogs could be the reservoir. The theory of canine origin of human kala azar was postulated by Nicolle as way back as in 1908 [27]. At that time dogs were found infected in nearly every endemic centre of human kala azar except India. Human and canine Kala Azar of the Mediterranean region is transmitted by the dog flea (*Ctenocephalus canis*) and perhaps also by the human flea (*Pulex irritans*) [28]. Small proportion of the dog fleas in many regions harbor a natural parasite *Herpetomonas ctenocephali* [29]. A possible case of human infection by *Herpetomonas* has been reported [30]. The sites of natural leptomonad infections in dog fleas are typically the hindgut and rectum, but in many insect groups, salivary glands and hemocoel have been reported to be infected [31]. Opportunistic infection with an insect trypanosomatid *Leptomonas pulexsimulantis*, a trypanosomatid found in the dog’s flea was diagnosed in an HIV positive patient presenting a clinical picture of visceral leishmaniasis co-infection [32]. The presence of *Leptomonas* of the dog’s flea in an HIV positive patient reinforces the idea that humans under immuno suppression conditions may be vulnerable to other insect trypanosomatids giving rise to clinical manifestations similar to leishmaniasis. Feces deposited by infected adult fleas are usually well supplied with amastigotes which retain their infectiousness after drying [30]. To facilitate transmission, these flagellar cysts, known as straphangers are capable of long term survival in adverse conditions. Dedet et al. [33] reported the first human case of *Leptomonas* infection in an HIV-infected patient. Amastigote forms were found to be present in the bone marrow aspirate of the HIV positive patient and these parasites grew in culture as promastigotes. However, infection in laboratory animals could not be established [34]. Monoxenous trypanosomatids can be pathogenic for human beings. Similar cases have also been reported [35], [36] where in HIV infected patients, *Leptomonas* parasites were detected with symptoms sometimes resembling those of visceral or cutaneous leishmaniasis. The present article also sustains the possibility of lower trypanosomatids infecting humans exists and should be considered by attending physicians. *Leptomonas* are opportunistic parasites with human infection possibly occurring *per os,* and so through our study we raise the question whether Kala Azar is leptomoniasis in some clinical cases in India? Due to morphological similarity and cross-reactivity with *Leishmania* species, human cases of infection with these lower trypanosomatids may have been underestimated. Or on the other hand, these clinical cases indeed represent *Leishmania-Leptomonas* co-infection. A *Leptomonas* of insect origin was highly susceptible to several standard trypanocides and leishmanicides *in vitro* and easily grown in defined media [37].

The important link then in the transmission of infection i.e. the sandfly vector, also needs to be questioned. Natural infection of *Phlebotomus longipalpis* by *Leptomonas*, in a focus of kala-azar has been reported [38]. Co-infection of *Leishmania* and *Leptomonas* in some sandflies in Nepal was confirmed by their rDNA signature [39]. Results of several studies have shown that *Phlebotomus argentipes*, the only known vector for *Leishmania donovani* in the Indian subcontinent, prefer to feed on both bovine and human blood [40]. Being a preferable host for *P. argentipes,* cattle was shown to play an undecided role in several epidemiological studies in the Indian subcontinent [41]. Chakravarty et al. [42] surveyed 64 cows along with dog, but could not find any amastigotes based on direct observation of smears from peripheral blood, liver, spleen, and bone marrow. On the other hand, *Leishmania* DNA was detected in several domestic animals including cattle from an endemic area in Nepal [43]. Studies conducted in Bangladesh to investigate the role of any domestic animal in VL transmission [44] shows that cattle are not a reservoir host for *L. donovani* despite its preference by *P. argentipes* as blood source. Ecological conditions should also be considered, changes in habitat associated with human development might create conditions suitable for establishment of anthroponotic cycles of infection with parasites which otherwise so far had been regarded as only monogenetic parasites of invertebrates.

Considering that *Leptomonas* was present in the splenic aspirate at time of collection, a question comes to mind that whether it could it be a hybrid profile (through genetic exchanges by recombination between the two species). *P. argentipus*, sand fly species might be transmitting both parasites? Co-infection of *Leishmania* and *Leptomonas* in some sandflies in Nepal has been established [39]. *Leishmania* parasites are capable of having a sexual cycle consistent with meiotic processes inside the insect vector [45]. Hybrid genotypes have been observed in field isolates involving most *Leishmania* species [46]–[49]. With the formation of genetic hybrids [50] new foci of disease may emerge as the hybrid progeny are transmitted to the mammalian vertebrate host by sandfly bites. Hybrid progenies within the vector host of *Leishmania major* have been established [51]. Is leishmaniasis in India caused by insect parasites?

For better disease management and healthcare, monitoring of *Leishmania* infection in sandflies is important to precise the eco-epidemiology of Kala-azar in India. Examination of domestic cattle for serological and molecular evidence of *Leishmania* infection in the VL endemic area in Bihar, India needs to be carried out. Parasites isolated from VL cases in India are routinely not typed, assuming that they are all *L. donovani* in contrast to other countries where typing is more systematically done. Investing in infrastructure to set up good typing centers and parasite banks needs to be undertaken. Ongoing clinical drug trials in India, are prone to result in dynamic selective pressures which may mould the genome of the parasites, therefore, profiling of VL in India using deep sequencing as a prospect of continuous surveillance of pathogenic parasites and their threat to public health should be greatly supported and encouraged by Indian government. We have established through this study the success of second generation sequencing technologies for building parasite whole genomes. This approach can now be adapted to studying local population genetics of the kinetoplastid parasites in India. Work is underway to assess and quantify the presence of *L. donovani* and *Leptomonas* directly from the human splenic aspirates using PCR.
